# A systematic review of crosswalks for converting patient-reported outcome measure scores in hip, knee, and shoulder replacement surgery

**DOI:** 10.2340/17453674.2024.41384

**Published:** 2024-09-13

**Authors:** Ilana N ACKERMAN, Sze-Ee SOH, Brian R HALLSTROM, Yi Ying FANG, Patricia FRANKLIN, Jörg LÜTZNER, Lina Holm INGELSRUD, for the International Society of Arthroplasty Registries Patient-Reported Outcome Measures Working Group

**Affiliations:** 1 School of Public Health and Preventive Medicine, Monash University, Melbourne, Australia; 2 School of Primary and Allied Health Care, Monash University, Melbourne, Australia; 3 Department of Orthopaedic Surgery, University of Michigan, Ann Arbor, USA; 4 Departments of Medical Social Sciences, Orthopedics, and Medicine (Rheumatology), Northwestern University Feinberg School of Medicine, Chicago, USA; 5 University Center of Orthopaedic, Trauma and Plastic Surgery, University Hospital Carl Gustav Carus, TU Dresden, Dresden, Germany; 6 Department of Orthopaedic Surgery, Copenhagen University Hospital, Hvidovre, Denmark

## Abstract

**Background and purpose:**

We aimed to systematically review studies of crosswalks for converting patient-reported outcome measure (PROM) scores used in joint replacement, and develop a database of published crosswalks.

**Methods:**

4 electronic databases were searched from January 2000 to May 2023 to identify studies reporting the development and/or validation of crosswalks to convert PROM scores in patients undergoing elective hip, knee, or shoulder replacement surgery. Data on study and sample characteristics, source and target PROMs, and crosswalk development and validation methods were extracted from eligible studies. Study reporting was evaluated using the Mapping onto Preference-based measures reporting Standards (MAPS) checklist.

**Results:**

17 studies describing 35 crosswalks were eligible for inclusion. Unidirectional crosswalks were available to convert hip-specific (Oxford Hip Score [OHS]) and knee-specific (Oxford Knee Score [OKS]) scores to the EQ-5D-3L/EQ-5D-5L. Similar crosswalks to convert disease-specific scores (WOMAC) to the EQ-5D-3L, EQ-5D-5L, and ICECAP-O Capability Index were identified. Bidirectional crosswalks for converting OHS and OKS to the HOOS-JR/HOOS-12 and KOOS-JR/KOOS-12, for converting WOMAC to the HOOS-JR/KOOS-JR, and for converting HOOS-Function/KOOS-Function to the PROMIS-Physical Function were also available. Additionally, crosswalks to convert generic PROM scores from the UCLA Activity Scale to the Lower Extremity Activity Scale in both directions were available. No crosswalks were identified for converting scores in shoulder replacement. Development methods varied with the type of target score; most studies used regression, item response theory, or equipercentile equating approaches. Reporting quality was variable, particularly for methods and results items, impacting crosswalk application.

**Conclusion:**

This is the first synthesis of published crosswalks for converting joint-specific (OHS, OKS, HOOS, KOOS), disease-specific (WOMAC), and generic PROMs scores (PROMIS-Physical Function, UCLA Activity Scale, Lower Extremity Activity Scale) used to assess joint replacement outcomes, providing a resource for data harmonization and pooled analysis. Crosswalks were developed using regression methods (9 studies), equipercentile equating methods (5 studies), a combination of equipercentile equating and item response theory methods (2 studies), and a combination of regression and equipercentile equating methods (1 study). A range of crosswalk validation approaches were adopted, including the use of external datasets, separate samples or subsets, follow-up data from additional time points, or bootstrapped samples. Efforts are needed to standardize crosswalk methodology and achieve consistent reporting.

Despite standardization efforts [1,2], there remains substantial international variation in patient-reported outcome measures (PROMs) used to assess joint replacement outcomes [3,4]. Most arthroplasty registries (64% of those recently surveyed [3]) administer a defined set of PROMs instruments and adding further measures has implications for responder burden, data completeness, and resourcing. In this context, methods that can facilitate data harmonization and support the pooled analysis of PROMs data from arthroplasty registries or clinical trials, without the need for additional data collection, are of potential value. Crosswalks are algorithms or scoring tables that enable a score from one PROM instrument to be converted (or “mapped”) to another PROM score. A variety of methods can be used to create crosswalks between PROMs, including equipercentile equating methods, item response theory methods and regression models (linear equating methods) [5-7]. While primary collection of PROMs data in the first instance will always be preferable, numerous crosswalks have been developed in recent years for PROMs instruments commonly used to assess joint replacement outcomes; for example, crosswalks between the HOOS or KOOS instruments and Oxford Hip or Knee Scores, respectively [8-10]. Regulatory requirements or jurisdictional norms require arthroplasty registries in some regions to collect certain PROMs. For example, in the United States, the KOOS-JR instrument is required to meet Center for Medicare and Medicaid Services requirements for knee replacements while in Europe the Oxford Knee Score is well accepted and used. Crosswalks may enable these groups to collaborate in ways otherwise not possible.

The extent of available crosswalks between generic, joint-specific or disease-specific PROMs has not been systematically examined and whether consistent approaches have been used to generate or report these crosswalks is unknown. A recent systematic review examined studies across 17 broad disease areas that mapped scores from quality of life or clinical measures to the EQ-5D health-related quality of life instrument [11]. This information was used to generate a database of crosswalks to support future cost-utility analyses. To the best of our knowledge, a similar resource has not been developed for published crosswalks pertaining to joint replacement outcomes. This systematic review aimed to synthesize contemporary evidence on crosswalks for converting PROMs scores used to assess hip, knee, and shoulder replacement outcomes. Specifically, we sought to understand:

1. What crosswalks have been developed for PROMs that are used to assess joint replacement outcomes?
2. What methods were used to develop and validate the crosswalks?

We also aimed to develop a freely available database of published crosswalks as an enduring resource for arthroplasty registries, clinicians, and researchers, to facilitate data harmonization efforts and dataset pooling and analysis.

## Methods

### Study design

This study was a systematic review, reported according to the Preferred Reporting Items for Systematic Reviews and Meta-Analyses (PRISMA) 2020 checklist [12]. The protocol was registered on the Open Science Framework (https://osf.io/b6fm7/), with a registered amendment to data extraction procedures in August 2023.

### Eligibility criteria

Studies were eligible for inclusion if they reported the development and/or validation of crosswalks to convert a PROMs instrument score (scale and/or summary score[s]) to another PROMs instrument score (scale and/or summary score[s]) in patients undergoing elective primary or revision hip, knee, or shoulder replacement. Studies involving patients undergoing unilateral, bilateral, total, or unicompartmental joint replacement were eligible. Studies involving mixed cohorts were eligible where the cohort included patients undergoing elective primary or revision joint replacement. We included studies reporting crosswalks for any PROMs instrument (including generic, disease-specific, and joint-specific instruments) in any language, where the study was reported in English, Danish, Norwegian, Swedish, or German. Studies focusing solely on patients undergoing joint replacement for trauma (for example, for treatment of fractures) or malignancy were ineligible. Literature reviews, conference abstracts, abstract-only publications, and grey literature were excluded.

### Search methods

We searched the Medline (OVID), EMBASE, CINAHL, and PsychINFO databases from January 1, 2000 to May 1, 2023. The search strategy combined 3 concepts: joint replacement surgery, PROMs instruments, and crosswalks. Search terms relating to PROMs instruments were based on University of Oxford PROM Group recommendations [13] and adapted for the context of this review. Search terms relating to crosswalks were informed by the search strategy used for a systematic review of crosswalks involving the EQ-5D instrument [11]. Key terms were mapped to subject headings, where possible, and exploded to include subheadings and related terms. Appropriate customized terms and Boolean operators were used for greater sensitivity. To broadly identify crosswalks relating to any instrument with relevance to the assessment of joint replacement outcomes, the search strategy was not restricted to specific PROMs instruments. The search strategy was initially developed for Medline and adapted for the other databases. A known set of relevant crosswalk studies in joint replacement settings [8-10] was used to test and refine the search strategy. The final search strategy and search yield is provided in Table S1 (see Supplementary data).

### Eligibility screening

2 review authors (INA and YYF) independently screened the titles and abstracts of the identified papers in Covidence to determine eligibility for inclusion. Full texts of potentially eligible papers were reviewed independently by 2 authors (INA and YYF) to confirm eligibility. Any discordance was resolved through consensus and arbitrated by a third author (SES), where needed.

### Additional reference searching

The reference lists of included studies were reviewed to identify further papers; a forward citation search was undertaken using Web of Science. Any potentially eligible studies were screened using methods described above.

### Assessment of reporting of key information

In the absence of a quality assessment or risk of bias tool designed specifically for crosswalk studies, the 23-item MApping onto Preference-based measures reporting Standards (MAPS) checklist [14] was used to summarize the reporting of key information in the included studies. As specified by the developers, the MAPS checklist aims to promote clarity, transparency, and completeness of reporting of mapping studies rather than assessing methodological quality. As the checklist was developed for crosswalks to utility scores, the wording of 3 items (items 1, 17, and 18) was revised for the purpose of this review to refer to any score or target measure. 2 review authors (SES and LHI) independently assessed the reporting of information in the included studies according to MAPS checklist items and discussed any disagreement to reach consensus. For each item, reporting was classified as “yes,” “partly” (as some checklist items include multiple elements), “no,” or “not applicable,” consistent with previous methods [11].

### Data extraction

Data was extracted using a standardized Excel form (Microsoft Corp, Redmond, WA, USA). 1 author (INA) extracted data from all included studies, and a second author (SES or LHI) independently extracted data from a randomly selected 6 studies to confirm accuracy. The following data was extracted:

- publication details: first author, year, journal, volume, issue, page numbers;
- study details: design, country, setting, research aim(s), research question(s);
- sample details: sample size for estimation and/or validation cohorts, proportion of patients undergoing joint replacement, primary/revision surgery, unicompartmental/total joint replacement, indication for surgery, descriptive statistics for age and sex;
- PROMs details: description of source and target PROMs (language, version, scoring range, scoring direction), data collection time point(s); and
- crosswalks details: crosswalk direction, development methods, validation methods, indicators of performance, accuracy, or error.

Where reported, we extracted electronic links and/or the published location of crosswalk tables, algorithms or other resources for converting PROMs scores.

### Data synthesis

All data is reported descriptively and no meta-analysis was planned given the nature of this data.

### Ethics, funding, data availability, and disclosures

Ethics approval was not required. Professor Ackerman was supported by a Monash University Faculty of Medicine, Nursing and Health Sciences Senior Postdoctoral Fellowship. Dr Hallstrom’s institution receives partial salary support from Blue Cross Blue Shield of Michigan for his work as Director of the Michigan Arthroplasty Registry Collaborative Quality Initiative (MARCQI). These institutions had no role in the study design, collection, analysis, and interpretation of data, in the writing of the manuscript, or in the decision to submit the manuscript for publication. All relevant data is available in the Tables, Supplementary data, and at the Open Science Framework as indicated. There are no conflicts of interests to declare. Complete disclosure of interest forms according to ICMJE are available on the article page, doi: 10.2340/17453674.2024.41384

## Results

### Study screening

**Figure 1:**
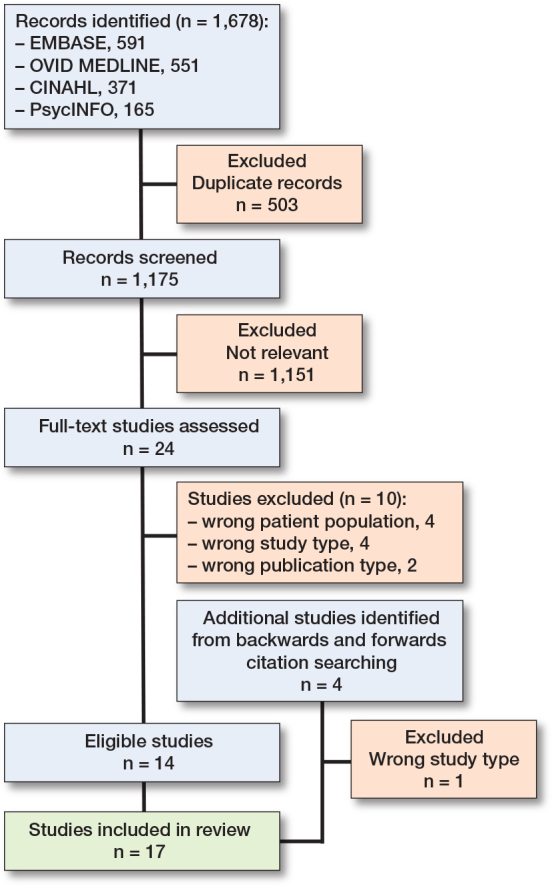
Flow diagram of included studies.

1,678 papers were identified from the database searches, including 503 duplicates, which were removed (Figure 1). A total of 1,175 papers underwent title and abstract screening, and 1,151 were excluded at this stage. Of the 24 full-text papers screened, 10 were excluded as they did not meet the eligibility criteria. Through backwards and forwards citation searching, an additional 4 papers were identified and screened; of these, 1 was excluded. In total, 17 studies met the inclusion criteria and were included in the review.

### Characteristics of the included studies

Of the 17 studies, 7 were conducted in the United States (US), 4 in the United Kingdom, 3 in Spain, 1 in Egypt, 1 in France, and 1 in Australia. The studies were predominantly conducted in hospital or arthroplasty registry settings (Table 1). Sample sizes ranged from 105 to 79,523 patients. 12 studies exclusively involved patients undergoing hip or knee replacement [8-10,15-23], 3 studies involved people with hip or knee osteoarthritis where a proportion underwent joint replacement [24-26], 1 study involved patients who were evaluated for hip replacement although some ultimately did not receive surgery [27], and 1 study involved patients with hip or knee osteoarthritis who required joint replacement [28]. Cohort characteristics are reported in Table 1.

**Table 1:** Characteristics of the included studies

Study	Country	Setting	Sample size	% undergoing joint replacement	Type of joint replacement	Dia-gnosis	Mean age (SD), years	% female/ women ^a^
Bilbao 2020 [24]	Spain	6 hospitals and 21 primary care centers	758	20 at 6 months	HR or KR (type NS)	OA	70 (11)	62
Clement 2022 [15]	UK	2 arthroplasty databases	Prediction cohort: 5,857 Validation cohort: 721	100	Primary TKR	NR	Prediction cohort: 71 (9) Validation cohort: 70 (9)	Prediction cohort: 62 Validation cohort: 56
Dakin 2013 [16]	UK	Randomized trial data plus PROMs dataset	Development cohort: 79,523 Validation cohort: 4,505	100	Primary or revision TKR	NR	NR	NR
Fawaz 2023 [25]	Egypt	NR	Estimation cohort: 456 Validation cohort: 115	Estimation sample: 13 (+ 24 ^b^ for TKR) Validation sample: 24 (+ 18 ^b^ for TKR)	TKR (primary or revision NS)	OA	Estimation cohort: 48 (13) Validation cohort: 49 (14)	Estimation cohort: 70 Validation cohort: 81
Fleisher 2022 [17]	USA	Single institution arthroplasty registry	HC: 4,649 KC: 3,751	100	Primary THR or TKR	OA	HC: 64 (11) KC: 67 (10)	HC: 52 KC: 60
Ghomrawi 2017 [18]	USA	2 arthroplasty registries from 1 institution	HC: 403 KC: 364	100	Primary THR or TKR	NR	HC: 66 (11) KC: 67 (9)	HC: 59 KC: 67
Heng 2021 [19]	USA	2 academic medical centers and 2 community hospitals	1,003	100	Primary TKR	98% OA	67 (8)	60
Heng 2022 [27]	USA	2 academic medical centers and 2 community hospitals	3,382	NR	Primary THR	OA	66 (11)	57
Martin-Fernandez 2020 [26]	Spain	Traumatology, rheumatology and primary care consultations in 3 areas	HC: 361 KC: 397	Baseline: 18 HR and 18 KR	THR or TKR (primary or revision NS)	OA	HC: 68 (12) KC: 71 (9)	HC: 47 KC: 30
Mitchell 2013 [28]	UK	Single hospital	105	100 requiring joint replacement	Primary HR or KR (type NS)	OA	70 (9)	49
Odum 2017 [20]	USA	8 high-volume orthopedic clinics	815	100	Primary THR	96% OA	67 (9)	58
Pinedo-Villanueva 2013 [21]	UK	4 acute National Health Service Trusts	1,719	100	Primary or revision THR	62% OA	70 (11)	64
Polascik 2020 [8]	USA	Hip registry (11 surgeons) and knee registry (8 surgeons)	HC: 486 KC: 340	100	Primary THR or resurfacing HR or primary TKR or UKR	NR	HC: 64 (11) KC: 66 (9)	HC: 48 KC: 54
Putman 2021 [9]	France	Tertiary care university hospital	500	100	Primary THR	OA or AVN	61 (14)	41
Soh 2022 [10]	Australia	National joint replacement registry	HC: 4,513 KC: 5,942	100	Primary THR or TKR	OA	HC: 66 (10) KC: 67 (9)	HC: 54 KC: 56
Tang 2022 [22]	USA	2 academic medical centers and 2 community hospitals	3,667	100	Primary TKR	NR	66 (11)	57
Wailoo 2014 [23]	Spain	15 hospitals in 3 regions	1,768	100	Primary HR or KR (type NS)	OA	69 (10)	Unclear

a Reported according to the terminology used in each study (sex/gender).

b Indicated but not further defined.

AVN: avascular necrosis; HC: hip cohort; HR: hip replacement; KC: knee cohort; KR: knee replacement; NR: not reported; NS: not specified; OA: osteoarthritis; THR: total hip replacement; TKR: total knee replacement; UKR: unicompartmental knee replacement.

### Reporting quality

Most studies reported key information of relevance in the title (16 studies), abstract (14 studies), and introduction (16 studies) (Table 2). However, the reporting of methods information was limited or absent in several studies; specifically, information pertaining to the external validation sample (partly reported or not reported in 6 studies), exploratory data analysis (partly reported or not reported in 8 studies), and the estimation of predicted scores or utilities (partly reported or not reported in 6 studies). Descriptive information was reported in 12 studies (and partly reported in the remaining 5 studies) and model uncertainty was infrequently reported (only 5 studies reported or partly reported this). All studies reported comparisons with previous studies and most (15 studies) reported limitations; the scope of applications was only partly reported in 6 studies.

**Table 2:** Reporting of key information according to the MAPS checklist

Checklist item ^a^	Reported?			Not applicable
	Yes	Partly	No	
Title and abstract				
1. Title	16	1		
2. Abstract	14	2	1	
Introduction				
3. Study rationale	16	1		
4. Study objective	16	1		
Methods				
5. Estimation sample	14	3		
6. External validation sample	1	4	2	10
7. Source and target measures	16	1		
8. Exploratory data analysis	9	5	3	
9. Missing data	12	2	3	
10. Modelling approaches	16	1		
11. Estimation of predicted scores or utilities	9	2	4	2
12. Validation methods	14	2	1	
13. Measures of model performance	12	2	1	2
Results				
14. Final sample size(s)	15	2		
15. Descriptive information	12	5		
16. Model selection	14			3
17. Model coefficients	8	2	1	6
18. Uncertainty	3	2	4	8
19. Model performance and face validity	15	2		
Discussion				
20. Comparison with previous studies	17			
21. Study limitations	15	2		
22. Scope of applications	11	6		
Other				
23. Additional information	17			

a See Petrou et al. [14] for a detailed description of the recommendation for each item.

Numbers represent totals across the 17 included studies.

The ‘not applicable’ option was used for studies where methods pertaining to the specific checklist item were not applied; for example, the reporting of external validation sample details was not applicable for studies that did not perform any external validation.

### Available crosswalks

**Figure 2:**
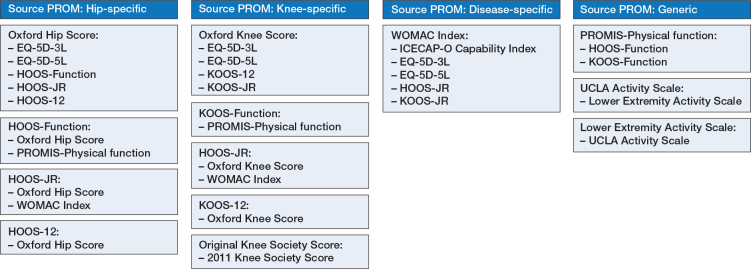
Overview of published crosswalks, by source instrument.

The Oxford Knee Score (6 studies), Oxford Hip Score (5 studies), and WOMAC Index (4 studies) were the most common source PROMs, while the EQ-5D instruments (either the 3-level or 5-level version) were the most common target PROM (7 studies) (Table 3). 10 studies reported unidirectional crosswalks (from the source PROM to the target PROM), while 7 studies reported bidirectional crosswalks. An overview of the published crosswalks, categorized by source PROM, is provided in Figure 2.

**Table 3:** Source and target instruments

Study	Details of source instrument(s)	Details of target instrument(s)	Crosswalk direction	Time point(s) used for crosswalk development
Bilbao 2020 [24]	WOMAC Index (Spanish version, 24 items)	EQ-5D-5L (Spanish version, 5 items; utility score derived from Spanish	Unidirectional	Baseline (preoperative for those who later underwent surgery)
Clement 2022 [15]	Oxford Knee Score (12 items)	EQ-5D-3L (5 items; utility score based on UK weights)	Unidirectional	Preoperative and 1-year postoperative
Dakin 2013 [16]	Oxford Knee Score (12 items)	EQ-5D-3L (5 items; utility score based on UK weights)	Unidirectional	Preoperative and up to 11 years postoperatively
Fawaz 2023 [25]	Oxford Knee Score (translated version NS), 12 items)	EQ-5D-5L (5 items; utility score based on weights from multiple countries)	Unidirectional	NS
Fleisher 2022 [17]	WOMAC Index (24 items)	HOOS-JR (6 items) KOOS-JR (7 items)	Bidirectional	Preoperative and 2 years postoperative
Ghomrawi 2017 [18]	UCLA Activity Scale (10 items)	Lower Extremity Activity Scale (18 items)	Bidirectional	Preoperative and 2 years postoperative
Heng 2021 [19]	KOOS-Function (17 items from KOOS-PS or full KOOS)	PROMIS-Physical function (version 1.0 SF 10a, 10 items)	Bidirectional	Preoperative and postoperative (median 62 days after surgery)
Heng 2022 [27]	HOOS-Function (5 items)	PROMIS-Physical function (version 10a, 10 items)	Bidirectional	Preoperative or postoperative (timing NS)
Martin-Fernandez 2020 [26]	Oxford Hip Score (Spanish version, 12 items) Oxford Knee Score (Spanish version, 12 items)	EQ-5D-5L (5 items; utility score based on Spanish population weights)	Unidirectional	Baseline (preoperative for those who later underwent surgery)
Mitchell 2013 [28]	WOMAC Index (24 items)	ICECAP-O Capability Index (5 capability attributes)	Unidirectional	Preoperative
Odum 2017 [20]	Original Knee Society Score (number of items not specified)	2011 Knee Society Score (number of items not specified)	Unidirectional	Either preoperative or post-operative (timing NS)
Pinedo-Villanueva 2013 [21]	Oxford Hip Score (12 items)	EQ-5D-3L (version inferred based on 243 health states; 5 items; utility score based on UK value set)	Unidirectional	Preoperative and 6-month postoperative
Polascik 2020 [8]	Oxford Hip Score (12 items) Oxford Knee Score (12 items)	HOOS-JR (6 items extracted from full HOOS) KOOS-JR (7 items extracted from full KOOS)	Bidirectional	Hip replacement: preoperative, 6 months, and 1–6 years post-operative Knee replacement: preoperative, 3 months, 6 months, and 1-year postoperative
Putman 2021 [9]	Oxford Hip Score (French version, 12 items)	HOOS-Function (French version, 5 items extracted from full HOOS) HOOS-JR (French version, 6 items extracted from full HOOS)	Bidirectional	Preoperative and postoperative (timing NS)
Soh 2022 [10]	Oxford Hip Score (12 items) Oxford Knee Score (12 items)	HOOS-12 (12 items) KOOS-12 (12 items)	Bidirectional	Preoperative and 6 months postoperative
Tang 2022 [22]	KOOS-Function (7 items)	PROMIS-Physical function (version 10a, 10 items)	Unidirectional	Preoperative or post-operative (timing NS)
Wailoo 2014 [23]	WOMAC Index (24 items)	EQ-5D-3L (number of items not specified; utility score based on UK tariffs)	Unidirectional	Preoperative, 3, 6, and 12 months postoperative

Information on the scoring range and direction of the source and target instruments, where reported, is provided in Supplementary file 2.

NS: not specified.

Multiple unidirectional crosswalks have been published to convert Oxford Hip Scores and Oxford Knee Scores to the EQ-5D-5L and EQ-5D-3L. According to Pinedo-Villanueva et al. [21], the mean EQ-5D-5L index score *(Ê_i_)* can be estimated from Oxford Hip Scores *(OHS_i_)* using the following formula:

*Ê_i_* = –0.070 + (0.022 × *OHS_i_*);

In order to estimate EQ-5D-3L utility scores from Oxford Knee Scores, a mapping algorithm fitted using multinomial logistic regression can be applied using Excel and Stata code available online [16]. Similar crosswalks to convert disease-specific measures such as the WOMAC to the EQ-5D-3L and EQ-5D-5L are also available. Using a 5-class mixture model, EQ-5D-3L utility scores can be reliably estimated using a calculator available online [23]. In contrast, 2 equivalent algorithms [24] can be used to map the WOMAC to the EQ-5D-5L (*Êi*):


$$\text{1. }{\hat{E}}_{i}=0.9516+0.0034*\frac{Pain^{2}}{100}-0.0044*\frac{Pain^{3}}{10^{4}}-0.0062*Function-0.0042*\frac{Pain*Function}{100};$$

$$\text{2. }{\hat{E}}_{i}=1.4162*B-0.4162;$$

where *B = e^A^/*(1 *+ e^A^*);

and $A=3.7265-0.0091*\frac{Pain^{3}}{10^{4}}-0.1145*Function+0.1633*\frac{Function^{2}}{100}-0.0948*\frac{Function^{3}}{10^{4}};$

Bidirectional crosswalks, mainly using equipercentile equating methods, to convert Oxford Hip Scores and Oxford Knee Scores to HOOS scores (including HOOS-Function, HOOS-JR, and HOOS-12 versions) and KOOS scores (including KOOS-Function, KOOS-JR and KOOS-12 versions) have also been published. Likewise, scoring tables to convert WOMAC scores to HOOS-JR and KOOS-JR are available. Details regarding the location of published crosswalk tables and links to online algorithms, calculators, and relevant code, where available, are outlined in Table S2 (see Supplementary data).

### Methods used to develop, assess, and validate the crosswalks

Substantial variation was identified in the methods used to develop the crosswalks. 9 studies exclusively used regression methods to develop the crosswalks while 5 studies exclusively used equipercentile equating methods (Table 4, see Appendix). Of the remainder, 2 studies used a combination of equipercentile equating and item response theory methods, and 1 study used a combination of regression and equipercentile equating methods. All the studies that sought to convert PROMs scores to EQ-5D-3L or EQ-5D-5L utility scores did so using regression methods. Among the studies that used regression models, the types of models varied substantially; for example, general linear models, tobit models, ordinary least squares, and quantile regression were used (Table 4, see Appendix). Across the included studies, crosswalk performance was assessed in a broad range of ways but was commonly evaluated by comparing actual and converted PROMs scores. Estimates of mean absolute error or root mean squared error were frequently reported; other indicators of crosswalk performance included intraclass correlation coefficients, Pearson’s or Spearman’s correlation coefficients, Bland–Altman limits of agreement, R-squared values, and the proportion of accurate (or inaccurate) score predictions. There was no consistency in crosswalk validation approaches, which varied widely across the studies (Table 4, see Appendix). While some studies used external datasets, separate samples, or follow-up data from additional time points to validate their crosswalks, other studies used subsets of their crosswalk development cohorts or bootstrapped samples (resampling with replacement from the original cohort).

**Table 4:** Methods used to develop, validate, and assess the crosswalks

Study	Development methods	Validation methods	Assessment of crosswalk performance
Bilbao 2020 [24]	3 approaches: general linear models tobit models beta models	6-month follow-up data was used to validate the preferred models	goodness-of-fit measures were used to select the preferred model per approach mean absolute error root mean squared error intra-class correlation coefficients
Clement 2022 [15]	Generalized linear regression analyses	Separate validation cohort was used; generalized linear regression analyses were used to predict utility scores for the modelling cohort and the validation cohort	difference between predicted and actual utility scores Bland-Altman limits of agreement
Dakin 2013 [16]	4 approaches: ordinary least squares regression generalized linear models with log link or gamma family fractional logistic models	External dataset was used to test the different mapping models with a full set of interaction terms	mean squared error mean absolute error
Fawaz 2023 [25]	4 approaches: cumulative probability for ordinal data penalized ordinal regression classification and regression trees ordinal random forest	External validation sample was used to determine accuracy	proportion of accurate predictions mean absolute errors mean squared error
Fleisher 2022 [17]	Equipercentile equating method using 70% of the full cohort	Preliminary validation using scores from the remaining 30% of the full cohort, then validated using 100 bootstrap samples with replacement for each crosswalk cohort	comparison of actual vs derived scores Spearman’s rank correlation coefficients root mean square errors
Ghomrawi 2017 [18]	Equipercentile equating method		comparison of mean actual vs converted scores comparison of standard response means for actual vs converted scores; area under the receiver operating characteristic curve (to assess discrimination ability)
Heng 2021 [19]	Equipercentile approach, with and without polynomial functions to smooth score distributions	Separate validation dataset not used	comparison of derived vs observed T-scores mean and standard deviation of differences between scores intraclass correlation coefficients Bland-Altman plots
Heng 2022 [27]	4 methods: equipercentile approach: with and without loglinear smoothing IRT-based methods: fixed item parameter calibration method separate parameter calibration method with Stocking-Lord constants	All available data points were used to validate the selected crosswalks in the non-surgical, pre-operative and post-operative subgroups using the Stocking-Lord approach	Pearson correlations between observed and linked T-scores mean and standard deviation of differences between T-scores root mean squared difference
Martin-Fernandez 2020 [26]	4 methods: ordinary least squares model tobit regression models generalized linear models beta regression models	Validation sample was used; intraclass correlation coefficients used to test relationship between predicted and observed values; mean absolute error and mean squared error used to assess predictions; Bland-Altman plots used to ascertain agreement between observed and predicted values	distribution of residuals coefficients of determination standard error of the coefficients intraclass correlation coefficients mean absolute errors mean squared error
Mitchell 2013 [28]	2 methods: ordinary least squares model multinomial logistic regression model	Follow-up data (1-year and 3-year postoperatively) were used to validate the predictions using ordinary least squares regression and multinomial logistic regression models; error was evaluated using mean absolute error, mean squared error and R-squared metrics	mean absolute error mean squared error goodness of fit
Odum 2017 [20]	Regression models	10-fold cross-validation where 10 different, equally sized data sets were randomly constructed from the overall dataset. Error between predicted and actual scores was measured for each of the 10 data sets	mean absolute error mean error proportion of observations with errors >10 points relative to the minimal clinically important difference Bland-Altman plots of agreement between observed and estimated scores
Pinedo-Villanueva 2013 [21]	4 methods: linear regression using continuous predictor data linear regression using categorical predictor data combined logistic/linear regression response mapping	internal validation using the estimation dataset external validation using a subset of the original cohort	difference between observed means and fitted means range of residuals % within 0.10 utility points of the observed score R-squared root mean square error mean absolute error linear correlation between observed and fitted utility scores
Polascik 2020 [8]	Equipercentile equating method		comparison of mean actual vs derived scores Spearman correlation coefficients for each timepoint and overall root mean square error for actual and derived score distributions
Putman 2021 [9]	5 methods: linear regression tobit regression quantile regression linear equating equipercentile equating	Cross-validation using 100 bootstrap iterations with ≤500 individuals randomly drawn with replacement from the study cohort	mean absolute error R-squared Kolmogorov-Smirnov distance
Soh 2022 [10]	Equipercentile equating method, with score distribution smoothed using log-linear models	Validation sample (a random one-third of each of the total hip replacement and total knee replacement cohorts) was used to evaluate crosswalk performance	comparison of mean actual vs derived scores Pearson correlation coefficients root mean square errors Bland-Altman plots
Tang 2022 [22]	5 methods: fixed-parameter calibration separate-parameter calibration with Stocking-Lord constants calibrated projection equipercentile methods with log-linear smoothing equipercentile methods with non-smoothing	Validated in a larger sample with the non-surgical, preoperative and post-operative groups, and in an external sample	mean difference standard deviation root mean squared deviation of score differences intraclass correlation coefficient for observed vs derived T-scores
Wailoo 2014 [23]	2 methods: mixture model linear model	None specified	mean absolute error root mean squared error Akaike Information Criterion Bayesian Information Criterion comparison of mean, median, standard deviation, minimum and maximum scores, proportion of highest scores

A database of published crosswalks, including details of study settings, samples, source, and target instruments and their scoring, and methods used for crosswalk development and validation, is provided in Supplementary data.

## Discussion

This systematic review is the first to synthesize the available international evidence on crosswalks for converting PROMs scores used to assess hip and knee replacement outcomes. We found 35 published crosswalks, with hip- or knee-specific PROMs being the most common source instruments. The identified crosswalks provide a relatively simple mechanism for converting PROMs scores where these are not directly collected, and for deriving preference-based utility scores from joint-specific or disease-specific scores. While primary data collection will always be preferable, these crosswalks can assist in aligning PROMs datasets to support pooled analysis and benchmarking, without expanding existing PROMs data collection or increasing responder burden.

Our review identified substantial variation in the ways that crosswalks are developed and validated, and inconsistencies in how crosswalk performance (or accuracy, with respect to converted versus actual scores) is evaluated. A range of regression models, item response theory methods, and equipercentile equating approaches were used in the included studies. The observed variation in statistical methods likely relates to differences in the types of target instruments and scores. For example, mapping PROMs scores to the EQ-5D-3L or EQ-5D-5L requires the use of regression methods to generate utility scores, rather than item response theory or equipercentile equating methods. However, among the crosswalk studies that utilized regression approaches, there was still substantial variation in the type of model and use of interaction terms. Various methodological approaches have their unique advantages and limitations. For example, although item response theory methods may be advantageous as different items from 2 PROMs instruments that measure the same construct can be calibrated on a shared underlying scale [5], they rely strongly on the assumption of unidimensionality. The assumption that one dominant trait is being measured would not be met by multidimensional PROMs instruments that concurrently assess joint pain, function, and/or quality of life. There is also some evidence (from joint replacement [19] and other clinical contexts [6]), to suggest that item response theory approaches can produce bias, compared with equipercentile equating methods. The advantage of regression models is that they allow equivalent scores to be estimated based on the observed mean and standard deviation for each PROM, but this assumes a linear relationship between scores on the 2 scales and may produce predicted scores beyond the range of possible scores. Finally, the equipercentile equating approach (which is based on non-parametric ranking methods) has been shown to result in less systematic bias [6]. However, this approach requires that the source and target instruments measure the same construct and are moderately correlated [29]. It also requires both scales to have sufficient range to reliably distinguish different percentiles [6]. The lack of consensus around the optimal statistical approach is evidenced by numerous included studies that used and compared multiple methods for crosswalk development [9,16,21,22,24-27]. Of the 3 included studies that used a combination of approaches, equipercentile equating methods showed either the lowest bias, smallest mean difference, or closest approximation of predicted to observed scores [9,22,27]. Where studies used multiple methods, the final crosswalks were often based on consideration of performance across a set of indicators, such as error estimates and prediction accuracy. We are unaware of any guidance for assessing crosswalk appropriateness (with respect to whether constructs or domains in the source and target instruments are sufficiently similar), although we note many crosswalks in this review were between instruments measuring closely related constructs such as pain and function.

There was considerable variation in the reporting of MAPS checklist items, particularly for the reporting of methods and results. While not an indicator of study quality per se, the incomplete reporting of methodological information limits the ability to decide whether a published crosswalk is appropriate for use by others. The importance of transparent reporting of crosswalk measurement and conversion errors has also been noted previously [30]. We are aware of efforts by ISPOR to develop guidance for conducting, appraising, and using mapping studies to convert scores from non-preference-based measures to utility values for health economic analysis [31]. We are not aware of similar initiatives that focus on non-preference-based measures as target instruments, including those used to evaluate pain and function. It is noteworthy that of the 17 studies included in this review, 8 were published in the past 3 years. These efforts reflect burgeoning interest in crosswalk development in the joint replacement field and underscore the need to establish consensus around statistical methods and consistent reporting to ensure a high-quality evidence base. We are also aware of accelerating US efforts to develop crosswalks between HOOS, KOOS, legacy joint-specific measures, and global pain and function measures of the Patient Reported Outcome Measurement Information System (PROMIS); these crosswalks are archived online [32].

Our joint replacement crosswalks database provides key details concerning study settings, sample characteristics, source and target PROMs, and crosswalk development and validation methods. To ensure practical value, the database provides links to available algorithms, code, and crosswalk tables that can be used to efficiently convert PROMs scores without technical input. We anticipate that this resource can be used within registry, clinical and research settings to allow group-level comparisons of previously collected PROMs data to promote data harmonization, enable pooled data analysis and support benchmarking efforts. However, given uncertainty in score predictions across the included studies, we do not recommend that crosswalks be used at the individual patient level. We suggest that potential users of crosswalks test these in their own setting (using published crosswalks that were derived from broadly similar patient samples, in terms of demographic and clinical characteristics). We also recognize the need for further research to evaluate the generalizability of developed crosswalks by validating the crosswalks in other cohorts.

This systematic review has also been important for identifying PROMs instruments for which multiple crosswalks already exist (to avoid future duplication of resources) and highlighting areas where new crosswalks are needed. A major gap is the absence of crosswalks to convert PROMs scores in the setting of shoulder replacement surgery. Through our database searches, we did identify 1 study that reported a crosswalk to map the Oxford Shoulder Score onto the EQ-5D utility index [33]; however, it used data from 4 clinical trials in shoulder arthroscopy, proximal humeral fracture, rotator cuff disease, and adhesive capsulitis. These trials either did not include shoulder replacement or included it only for the treatment of fractures. Given marked growth in shoulder replacement populations internationally [34-36] and the potential benefits of data pooling (given the relatively small size of shoulder cohorts in arthroplasty registries), the development of crosswalks for this patient group should be considered for situations where expanding the collection of PROMs data is not feasible. This could include crosswalks between the Oxford Shoulder Score and American Shoulder and Elbow Surgeons Score, which are used by multiple arthroplasty registries [3].

### Strengths

Key strengths include the use of accepted methods for searching, screening, data extraction, and evaluation of reporting. We considered all types of PROMS instruments with relevance to joint replacement and included studies involving people undergoing elective primary, revision, partial, or total joint replacement. The review was not restricted to English-version PROMs instruments and the included studies came from 6 countries.

### Limitations

We also acknowledge the limitations. We focused on the published peer-reviewed literature and recognize other crosswalks may be available from websites or reports. Other studies have generated crosswalks with potential relevance to joint replacement outcome assessment (for example, crosswalks from the SF-12 to the EQ-5D [37, 38]); however, they utilized general population samples and did not meet our inclusion criteria. Finally, we did not intend to assess the quality or appropriateness of crosswalk development and validation methods as there are currently no criteria to enable this.

### Conclusions

This systematic review identified 17 studies describing 35 crosswalks for converting PROMs scores in joint replacement. These include crosswalks to convert hip-specific (Oxford Hip Score and HOOS variants), knee-specific (Oxford Knee Score and KOOS variants), disease-specific (WOMAC), and generic PROM scores (PROMIS-Physical Function, UCLA Activity Scale, and Lower Extremity Activity Scale), with the EQ-5D (3L or 5L) being the most common target instrument. Crosswalks were developed using a range of approaches, including regression methods, equipercentile equating methods, item response theory methods, or a combination of these. Crosswalk validation methods varied across the studies and included the use of external datasets, separate samples or subsets, follow-up data from additional time points, or bootstrapped samples. We did not find any crosswalks for converting PROM scores in shoulder replacement and this is a notable area for future research. While derived scores should never replace collected scores and crosswalks should be tested in local contexts, our database of published crosswalks represents a new resource for arthroplasty registries and researchers where modifying existing PROMs data collection procedures is not feasible. This resource can be updated as new crosswalks emerge and as further crosswalk validation studies are published. Efforts to standardize crosswalk development and validation and report these processes consistently will be helpful for facilitating future data harmonization.

### Supplementary data

Tables S1 and S2 and a detailed database of published crosswalks are available as supplementary data on the article page, doi: 10.2340/17453674.2024.41384

## Supplementary Material





## Notes

INA: conceptualization, methodology, data curation, formal analysis, investigation, writing – original draft, writing – review and editing. SES and LHI: conceptualization, methodology, data curation, formal analysis, investigation, writing – review and editing. BRH: conceptualization, methodology, writing – review and editing. YYF: data curation, formal analysis, investigation, writing – review and editing. PF: conceptualization, methodology, writing – review and editing. JL: methodology, writing – review and editing.

The authors would like to thank Micheline Turnau from the Canadian Institute for Health Information for her input into the development of the systematic review protocol and review of the manuscript.

Handling co-editors: Jeppe Vejlgaard Rasmussen and Robin Christensen

*Acta* thanks Alma B Pedersen and Anne Mørup-Petersen for help with peer review of this manuscript.
