# Supplementary material for: A systematic review of crosswalks for converting patient-reported outcome measure scores in hip, knee, and shoulder replacement surgery
Source: Acta Orthop. 2024 Sep 13;95:512–23. doi: 10.2340/17453674.2024.41384 (PMC11494241; doi:10.2340/17453674.2024.41384)
Supplement: Supplementary file 1 [file ActaO-95-41384-s1.pdf]

A systematic review of crosswalks for converting patient-reported outcome measure scores in hip, knee, and shoulder replacement surgery

Ilana Ackerman, Sze-Ee Soh, Brian Hallstrom, Yi Ying Fang, Patricia Franklin,

Jörg Lützner and Lina Holm Ingelsrud

Supplementary file

Table S1. Search strategy and yield, by database

	Ovid MEDLINE	Embase	PsycInfo	CINAHL
1	exp Arthroplasty, Replacement, Hip/ or exp Arthroplasty, Replacement/ or exp Arthroplasty, Replacement, Shoulder/ or exp Arthroplasty, Replacement, Knee/	exp Arthroplasty, Replacement, Hip/ or exp Arthroplasty, Replacement/ or exp Arthroplasty, Replacement, Shoulder/ or exp Arthroplasty, Replacement, Knee/	(arthroplast* or replacement?).mp or exp Hips/ or exp Knee/ or shoulder.mp	(MH "Arthroplasty+") OR (MH "Arthroplasty, Replacement, Shoulder+") OR (MH "Arthroplasty, Replacement, Knee+") OR (MH "Arthroplasty, Replacement, Hip") OR (MH "Arthroplasty, Replacement+")
2	(mapping or map or mapped or crosswalk? or cross-walk? or equate or equating or agreement? or link or linking or bi?directional or convert* or "transfer to utility").mp.	(mapping or map or mapped or crosswalk? or cross-walk? or equate or equating or agreement? or link or linking or bi?directional or convert* or "transfer to utility").mp.	(mapping or map or mapped or crosswalk? or cross-walk? or equate or equating or agreement? or link or linking or bi?directional or convert* or "transfer to utility").mp.	mapping OR map OR mapped OR cross-walk* OR crosswalk* OR "transfer to utility" OR “indirect utility”
3	(PROM or HR-PRO or HRPRO or HRQL or HRQoL or QL or QoL or quality of life or life quality or patient-reported outcome measure* or pain or (health index* or health indices or health profile*) or health status or ((disability or function* or subjective or utility* or wellbeing or well being) adj2 (index* or indices or instrument? or measure? or questionnaire? or profile? or scale? or score? or status or survey?))).mp.	(PROM or HR-PRO or HRPRO or HRQL or HRQoL or QL or QoL or quality of life or life quality or patient-reported outcome measure* or pain or (health index* or health indices or health profile*) or health status or ((disability or function* or subjective or utility* or wellbeing or well being) adj2 (index* or indices or instrument? or measure? or questionnaire? or profile? or scale? or score? or status or survey?))).mp.	(PROM or HR-PRO or HRPRO or HRQL or HRQoL or QL or QoL or quality of life or life quality or patient-reported outcome measure* or pain or (health index* or health indices or health profile*) or health status or ((disability or function* or subjective or utility* or wellbeing or well being) adj2 (index* or indices or instrument? or measure? or questionnaire? or profile? or scale? or score? or status or survey?))).mp.	PROM or HR-PRO or HRPRO or HRQL or HRQoL or ql or QoL or “quality of life” or “life quality” or “patient-reported outcome measure*” or pain or “health index*” or “health indices” or “health profile*” or “health status” or ((disability or function* or subjective or utility* or wellbeing or “well being”) N2 (index* or indices or instrument# or measure# or questionnaire# or profile# or scale# or score# or status or survey#))
4	1 and 2 and 3	1 and 2 and 3	1 and 2 and 3	1 and 2 and 3
5	limit 4 to yr=”2000-Current”	limit 4 to yr=”2000-Current”	limit 4 to yr=”2000-Current”	Limiters - Published Date: 20000101-20231231
**Yield**	**551**	**591**	**165**	**371**

Table S2. Tools for using the available crosswalks

Study	Source PROM	Target PROM	Online tool	Crosswalk tables in published paper
Bilbao 2020	WOMAC Index	EQ-5D-5L	Syntax in R (Appendix, Table 3S). Available from: https://doi.org/10.1016/j.jval.2019.09.2755	
Clement 2022	Oxford Knee Score	EQ-5D-3L		
Dakin 2013	Oxford Knee Score	EQ-5D-3L	Stata command and Excel spreadsheet for converting scores. Available from: https://www.herc.ox.ac.uk/downloads/downloads-supporting-material-1/mapping-analyses-to-estimate-eq-5d-utilities-and-responses-based-on-oxford-knee-score	
Fawaz 2023	Oxford Knee Score	EQ-5D-5L		
Fleisher 2022	WOMAC Index	HOOS-JR<br>KOOS-JR	Crosswalk tables (Appendix Tables 1-2). Available from: https://www.sciencedirect.com/science/article/pii/S0883540321008445#appsec1	
Ghomrawi 2017	UCLA Activity Scale	Lower Extremity Activity Scale		
Heng 2021	KOOS-Function	PROMIS-Physical function		Tables 2 and 3
Heng 2022	HOOS-Function	PROMIS-Physical function		Tables 5 and 6
Martin-Fernandez 2020	Oxford Hip Score<br>Oxford Knee Score	EQ-5D-5L		
Mitchell 2013	WOMAC Index	ICECAP-O Capability Index		
Odum 2017	Original Knee Society Score	2011 Knee Society Score	Spreadsheet for converting scores (Supplementary file). Available from: https://journals.lww.com/clinorthop/Fulltext/2017/01000/Can_Original_Knee_Society_Scores_Be_Used_to.29.aspx	
Pinedo-Villanueva 2013	Oxford Hip Score	EQ-5D		
Polascik 2020	HOOS-JR<br>KOOS-JR	Oxford Hip Score<br>Oxford Knee Score		Table 4
Putman 2021	Oxford Hip Score	HOOS-Function<br>HOOS-JR<br>HOOS-12		Tables 4 and 5
Soh 2022	Oxford Hip Score<br>Oxford Knee Score	HOOS-12<br>KOOS-12		Tables 2 and 5
Tang 2022	KOOS-Function	PROMIS-Physical function		Table 3
Wailoo 2014	WOMAC Index	EQ-5D	EQ-5D calculator (Additional file 1). Available from: https://hqlo.biomedcentral.com/articles/10.1186/1477-7525-12-37#Sec8	
